# Effect of Molecular Architecture on Associating Behavior of Star-Like Amphiphilic Polymers Consisting of Plural Poly(ethylene oxide) and One Alkyl Chain

**DOI:** 10.3390/polym13030460

**Published:** 2021-01-31

**Authors:** Daisuke Kugimoto, Aoi Taniguchi, Masaki Kinoshita, Isamu Akiba

**Affiliations:** Department of Chemistry and Biochemistry, The University of Kitakyushu, Kitakyushu 8080135, Japan; u3maa004@eng.kitakyu-u.ac.jp (D.K.); x6maa010@eng.kitakyu-u.ac.jp (A.T.); u3maa003@eng.kitakyu-u.ac.jp (M.K.)

**Keywords:** polymer micelles, star-like polymer, small-angle X-ray scattering

## Abstract

Associating behavior of star-like amphiphilic polymers consisting of two or three poly(ethylene oxide) (PEO) chains and one stearyl chain (C18) was investigated. Although the aggregation number (*N_agg_*) of linear analogue of amphiphilic polymers monotonically decreased with increasing number-average molecular weight of PEO (*M_n_*_,PEO_), the *N_agg_* of micelles of star-like amphiphilic polymers with *M_n_*_,PEO_ = 550 g/mol was smaller than that with *M_n_*_,PEO_ = 750 g/mol, whereas that with *M_n_*_,PEO_ ≥ 750 g/mol showed general *M_n_*_,PEO_ dependence. Small-angle X-ray scattering analyses revealed that the occupied area of one PEO chain on the interface between hydrophobic core and corona layer in the micelles of star-like polymers was much narrower than that in the linear amphiphilic polymers. This result indica ted the PEO chains of star-like polymers partially took unfavorable conformation near the core–corona interface in polymer micelles. The effect of local conformation of PEO chains near the interface on the associating behavior became significant as *M_n_*_,PEO_ decreased. Therefore, in polymer micelles of star-like amphiphilic polymers containing PEO with *M_n_*_,PEO_ = 550 g/mol, the enlargement of occupied area of PEO on the core–corona interface should be caused to avoid the formation of unfavorable conformations of partial PEO chains, resulting in a decrease in *N_agg_*s.

## 1. Introduction

Amphiphilic polymers, such as block copolymers composed of hydrophobic and hydrophilic polymer chains and hydrophobically modified water-soluble polymers, spontaneously assemble into polymer micelles consisting of a hydrophobic core and hydrated corona in aqueous solution [1]. Because polymer micelles have a suitable size in the range of several nm to several 100 nm, accumulate in tumors, can uptake hydrophobic compounds as anticancer drugs, and show a “stealth effect” which is a function that is not captured by a reticuloendothelial system such as a liver, they are attracting much attention in drug delivery systems as drug vehicles [2,3,4,5,6].

Such polymer micelles can have various shapes, sizes and radial density distributions depending on the length of hydrophobic and hydrophilic polymer chains, hydrophobicity, excluded volume interactions between the hydrophilic polymer chains in swollen corona and so on [7,8,9]. In the case of polymer micelles, structures are generally controlled by tuning chain lengths of hydrophobic and hydrophilic chains, and their ratios [7,8,9]. However, because polymers have a high degree of freedom in molecular architecture, topological feature of amphiphilic polymers also have significant effects on structures of polymer micelles because it should have a significant effect on excluded volume interactions and conformation of polymer chains [10,11,12]. When a star-like amphiphilic polymer, in which the ends of several polymer chains are connected at one point, is used as a component of a polymer micelle, the number density or occupied area of polymer chains at the interface between hydrophobic core and corona layer that determines the excluded volume interactions between polymer chains [13,14,15] should depend on the number of chains participating in the star-like polymer. Consequently, the associating behavior of the star-like amphiphilic polymers should strongly depend on the number of polymer chains. In addition, this effect is considered to differ depending on the chain length. Thus, in this study, we focus on the effects on the number and chain length of hydrophilic chains on the associating behavior of star-like amphiphilic polymers consisting of one hydrophobic alkyl chain and plural poly(ethylene oxide) (PEO) as a hydrophilic chain.

## 2. Materials and Methods

### 2.1. Reagents

Stearoyl chloride, 1,3-dibromo-2-propanol, pentaerythritol tribromide and sodium hydride (NaH) were purchased from Tokyo Chemical Industry Co. Ltd. (Tokyo, Japan). Poly(ethylene oxide) monomethyl ethers (PEO) with 550, 750, 2000, and 5000 g/mol of number-average molecular weight *M*_n_ were purchased from Sigma-Aldrich Co. Ltd. (St. Louis, MO, USA). According to *M_n_* of PEO (*M_n_*_,PEO_), PEO are described as PEO550, PEO750, PEO2k and PEO5k, respectively. Pyridine and dehydrated tetrahydrofuran (THF) were purchased from Fujifilm Wako Chemicals Co. Ltd. (Tokyo, Japan). All reagents were used as obtained. Star-like amphiphilic polymers consisting of plural PEO chains and one stearyl chain were synthesized by the methods shown below. The amphiphilic polymers are denoted as (PEO*x*)*_y_*-C18, where *x* and subscription *y* are *M_n_*_,PEO_ and number of PEO chains in a star-like polymer, respectively.

### 2.2. Synthesis of 1,3-dibromo-2-propyl Stearate (1)

1,3-dibromo-2-propanol (1.17 g, 5.40 mmol) was dissolved in THF in a round-bottom flask capped with a rubber septum under dry nitrogen atmosphere. Pyridine (0.42 g, 5.40 mmol) was added into the flask and stirred at ambient temperature. Then, the solution was allowed to cool by ice-cold water bath. Stearoyl chloride (1.5 g, 4.90 mmol) was added dropwise to the solution. After the solution was stirred at ambient temperature for 24 h, precipitate was filtered off. Then, the resulting solution was extracted with water and hexane. The organic layer was dried with MgSO_4_ and evaporated to dryness under reduced pressure. The crude product was purified by column chromatography, eluting with hexane to give **1** as a white solid: yield 32%; ^1^H NMR (CDCl_3_) δ 5.05 (m, 1H, CO_2_–C*H*–), 3.52 (s, 4H, Br–C*H*_2_–), 2.26(t, 2H, OCO–C*H*_2_–), 1.58 (m, 2H, OCO–CH_2_–C*H*_2_–), 1.35 (m, 28H, CH_2_–C*H*_2_–CH_2_), 0.82 (t, 3H, CH_2_–C*H*_3_).

### 2.3. Synthesis of 3-bromo-2,2-dibromomethyl-1-propyl Stearate (2)

Pentaerythritol tribromide (2.3 g, 6.9 mmol) was dissolved in THF in a round-bottom flask capped with a rubber septum under dry nitrogen atmosphere. Pyridine (0.54 g, 6.9 mmol) was added to the flask and stirred at ambient temperature. Then, the solution allowed to cool by ice-cold water bath. Stearoyl chloride (2.1 g, 6.9 mmol) was added dropwise to the solution. After the solution was stirred at ambient temperature for 24 h, precipitate was filtered off. Then, the solution was extracted with water and hexane. The organic layer was dried with MgSO_4_ and evaporated to dryness under reduced pressure. The crude product was purified by column chromatography, eluting with hexane to give **2** as a white solid: yield 33%; ^1^H NMR (CDCl_3_) δ 4.12 (s, 2H, CO_2_–C*H*_2_), 3.11 (s, 6H, Br–C*H*_2_–), 2.31 (t, 2H, OCO–C*H*_2_–), 1.62 (m, 2H, OCO-CH_2_–C*H*_2_–), 1.35 (m, 28H, CH_2_–C*H*_2_–CH_2_), 0.82 (t, 3H, CH_2_–C*H*_3_).

### 2.4. Synthesis of (PEOx)_1_-C18

In the round-bottom flask, PEO was dissolved in THF. After the flask was capped with a rubber septum and filled with N_2_, pyridine (1 equiv. to PEO) was added into the flask and stirred at ambient temperature. Then, the solution allowed to cool by ice-cold water bath. Stearoyl chloride (1 equiv. to PEO) was added dropwise to the solution. The reaction mixture was stirred overnight at ambient temperature. After precipitate was filtered off, the resulting solution was extracted with water and hexane, and aqueous layer was dialyzed for 5 days to remove unreacted PEG. The solution was dried by freeze drying to give (PEO*x*)_1_-C18 as a white powder.

*(PEO550)_1_-C18.* Yield = 26 %; ^1^H-NMR (CDCl_3_) δ 4.14 (m, 2H COO–C*H*_2_), 3.76 (m, 2H, COO–CH_2_–C*H*_2_–O), 3.60 (m, 45H, O–C*H*_2_–C*H*_2_–O), 3.40 (s, 3H, O–C*H*_3_), 2.28 (m, 2H, OCO–C*H*_2_), 1.64 (m, 2H, OCO–CH_2_–C*H*_2_), 1.24 (m, 28H, CH_2_–C*H*_2_–CH_2_), 0.85 (t, 3H CH_2_–C*H*_3_).

*(PEO750)_1_-C18.* Yield = 32 %; ^1^H-NMR (CDCl_3_) δ 4.18 (m, 2H COO–C*H*_2_), 3.75 (m, 2H, COO–CH_2_–C*H*_2_–O), 3.60 (m, 62H, O–C*H*_2_–C*H*_2_–O), 3.38 (s, 3H, O–C*H*_3_), 2.26 (m, 2H, OCO–C*H*_2_), 1.64 (m, 2H, OCO–CH_2_–C*H*_2_), 1.22 (m, 28H, CH_2_–C*H*_2_–CH_2_), 0.83 (t, 3H CH_2_–C*H*_3_).

*(PEO2k)_1_-C18.* Yield = 22 %; ^1^H-NMR (CDCl_3_) δ 4.15 (m, 2H COO–C*H*_2_), 3.76 (m, 2H, COO–CH_2_–C*H*_2_), 3.62 (m, 180H, O–C*H*_2_–C*H*_2_–O), 3.40 (s, 3H, O–C*H*_3_), 2.30 (m, 2H, OCO–C*H*_2_), 1.65 (m, 2H, OCO–CH_2_–C*H*_2_), 1.24 (m, 28H, CH_2_–C*H*_2_–CH_2_), 0.85 (t, 3H CH_2_–C*H*_3_).

*(PEO5k)_1_-C18.* Yield = 17 %; ^1^H-NMR (CDCl_3_) δ 4.14 (m, 2H COO–C*H*_2_), 3.76 (m, 2H, COO–CH_2_–C*H*_2_–O), 3.60 (m, 440H, O–C*H*_2_–C*H*_2_–O), 3.38 (s, 3H, O–C*H*_3_), 2.28 (m, 2H, OCO–C*H*_2_), 1.64 (m, 2H, OCO–CH_2_–C*H*_2_), 1.24 (m, 28H, CH_2_–C*H*_2_–CH_2_), 0.85 (t, 3H CH_2_–C*H*_3_).

### 2.5. Synthesis of (PEOx)_2_-C18

**Scheme 1:**
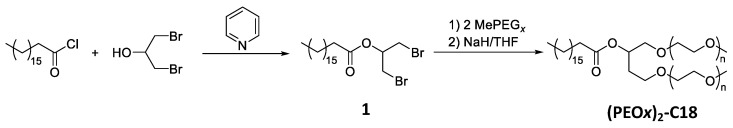
Synthesis of (PEO*x*)_2_-C18.

(PEO*x*)_2_-C18s were synthesized according to Scheme 1. NaH was dispersed in THF in a two-neck flask, and the flask was filled with N_2_. PEO (1 equiv. to NaH) was dissolved in THF in another flask under N_2_ atmosphere, and then the THF solution was added dropwise to the two-neck flask. The reaction mixture was stirred at ambient temperature for 30 min to be completely deprotonated. **1** (0.45 equiv. to PEO) was dissolved in THF, and then added dropwise to the two-neck flask. The reaction mixture was refluxed for 5 days. After precipitates were filtered off, the filtrate was extracted with water and hexane and aqueous layer was dialyzed to remove unreacted PEO. The solution was dried by freeze driying to give (PEO*x*)_2_-C18 as a white powder.

*(PEO550)_2_–C18.* Yield = 21 %; ^1^H-NMR (CDCl_3_) δ 4.19 (m, 1H COO–C*H*), 3.82–3.55 (m, 110H, CH–C*H*_2_-O and O–C*H*_2_-C*H*_2_–O), 3.38 (s, 6H, CH_2_-O–C*H*_3_), 2.25 (m, 2H, OCO-C*H*_2_–CH_2_), 1.62 (m, 2H, OCO-CH_2_–C*H*_2_-CH_2_), 1.24 (m, 28H, CH_2_–C*H*_2_–CH_2_), 0.83 (t, 3H CH_2_–C*H*_3_).

*(PEO750)_2_-C18.* Yield = 36 %; ^1^H-NMR (CDCl_3_) δ 4.20 (m, 1H COO–C*H*), 3.82–3.55 (m, 142H, CH–C*H*_2_-O and O–C*H*_2_–C*H*_2_–O), 3.40 (s, 6H, CH_2_-O–C*H*_3_), 2.28 (m, 2H, OCO–C*H*_2_–CH_2_), 1.60 (m, 2H, OCO–CH_2_–C*H*–CH_2_), 1.23 (m, 28H, CH_2_–C*H*_2_-CH_2_), 0.86 (t, 3H CH_2_–C*H*_3_).

*(PEO2K)_2_–C18.* Yield = 42 %; ^1^H-NMR (CDCl_3_) δ 4.20 (m, 1H COO-C*H*), 3.82–3.55 (m, 370H, CH–C*H*_2_–O and O–C*H*_2_–C*H*_2_–O), 3.40 (s, 6H, CH_2_–O–C*H*_3_), 2.26 (m, 2H, OCO–C*H*_2_–CH_2_), 1.62 (m, 2H, OCO–CH_2_-C*H*_2_–CH_2_), 1.24 (m, 28H, CH_2_–C*H*_2_–CH_2_), 0.85 (t, 3H CH_2_–C*H*_3_).

*(PEO5K)_2_–C18.* Yield = 43 %; ^1^H-NMR (CDCl_3_) δ 4.18 (m, 1H COO–C*H*), 3.82–3.55 (m, 920H, CH–C*H*_2_–O and O–C*H*_2_-C*H*_2_–O), 3.40 (s, 6H, CH_2_–O–C*H*_3_), 2.25 (m, 2H, OCO-C*H*_2_–CH_2_), 1.61 (m, 2H, OCO–CH_2_–C*H*_2_–CH_2_), 1.24 (m, 28H, CH_2_–C*H*_2_–CH_2_), 0.83 (t, 3H CH_2_–C*H*_3_).

### 2.6. Synthesis of (PEOx)_3_-C18

**Scheme 2:**
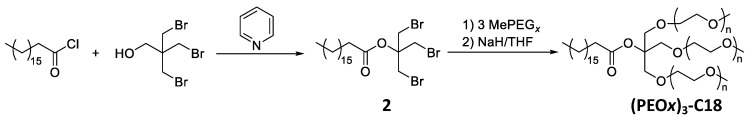
Synthesis of (PEO*x*)_3_-C18.

(PEO*x*)_3_-C18s were synthesized according to Scheme 2. NaH was dispersed in THF in a two-neck flask, and the flask was filled with N_2_. PEO (1 equiv. to NaH) was dissolved in THF in another flask under N_2_ atmosphere, and then the THF solution was added dropwise to the two-neck flask. The reaction mixture was stirred at ambient temperature for 30 min to be completely deprotonated. **2** (0.3 equiv. to PEO) was dissolved in THF, and then added dropwise the two-neck flask. The reaction mixture was refluxed for 5 days. After precipitates were filtered off, the filtrate was extracted with water and hexane and aqueous layer was dialyzed to remove unreacted PEO. The solution was dried by freeze drying to give (PEO*x*)_3_-C18 as a white powder.

*(PEO550)_3_–C18.* Yield 46%; ^1^H-NMR (CDCl_3_) δ 4.12 (s, 2H, CO_2_–C*H*_2_), 3.75–3.50 (s, 162H, C–C*H*_2_–O and O–C*H*_2_–C*H*_2_–O), 2.28 (t, 2H, OCO–C*H*_2_–), 1.78 (m, 2H, OCO–CH_2_–C*H*_2_–), 1.26 (m, 28H, CH_2_–C*H*_3_–CH_2_), 0.82 (t, 3H, CH_2_–C*H*_3_).

*(PEO750)_3_–C18.* Yield 38%; ^1^H NMR (CDCl_3_) δ 4.11 (s, 2H, CO_2_–C*H*_2_), 3.75–3.50 (s, 210H, C–C*H*_2_–O and O–C*H*_2_–C*H*_2_–O), 2.28 (t, 2H, OCO–C*H*_2_–), 1.83 (m, 2H, OCO–CH_2_–C*H*_2_–), 1.25 (m, 28H, CH_2_–C*H*_2_–CH_2_), 0.84 (t, 3H, CH_2_–C*H*_3_).

*(PEO2K)_3_–C18.* Yield 33%; ^1^H-NMR (CDCl_3_) δ 4.12 (s, 2H, CO_2_–C*H*_2_), 3.80–3.50 (s, 560H, C–C*H*_2_–O and O–C*H*_2_–C*H*_2_–O), 2.32 (t, 2H, OCO–C*H*_2_–), 1.84 (m, 2H, OCO–CH_2_–C*H*_2_–), 1.25 (m, 28H, CH_2_–C*H*_2_–CH_2_), 0.85 (t, 3H, CH_2_–C*H*_3_).

(PEO5K)_3_–C18. Yield 35%; ^1^H-NMR (CDCl_3_) δ 4.15 (s, 2H, CO_2_–C*H*_2_), 3.80–3.50 (s, 1380H, C–C*H*_2_–O and O–C*H*_2_–C*H*_2_–O), 2.29 (t, 2H, OCO–C*H*_2_–), 1.80 (m, 2H, OCO–CH_2_–C*H*_2_–), 1.25 (m, 28H, CH_2_–C*H*_2_–CH_2_), 0.85 (t, 3H, CH_2_–C*H*_3_).

### 2.7. Preparation of Polymer Micelles

Amphiphilic polymers were dispersed in nanopure water, which was prepared by Barnstead Nanopure System, at a desired concentration. The solutions were homogenized by using a bath-type ultrasonic homogenizer. The resulting micelle solutions were further clarified by centrifugation and filtrated through a membrane filter with 0.2 μm of pore size.

### 2.8. Characterization

**Figure 1:**
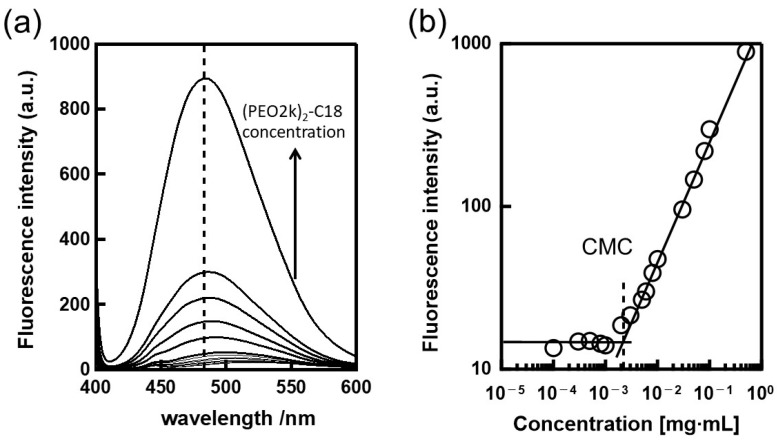
(**a**) Change in fluorescence spectrum of sodium 8-anilino-1-naphthalene sulfonate (ANS-Na) in aqueous (PEG2k)_2_-C18 solution with change of (PEG2k)_2_-C18 concentration. (**b**) (PEG2k)_2_-C18 concentration dependence of fluorescence intensity at 490 nm (*I*_490nm_). Critical micelle concentration (CMC) is defined as the concentration at which the slope of concentration dependence of *I*_490nm_ drastically changes.

^1^H-NMR spectra were recorded on a JEOL JNM ECP-500 NMR spectrometer (Tokyo, Japan). Chemical shifts were referenced to TMS and CHCl_3_ slightly contained in CDCl_3_. Gel permeation chromatography (GPC) eluted with THF was conducted on a system equipped with an isocratic pump model PU-4180, a differential refractometer model RI-4030 (JASCO, Tokyo, Japan), and a PSgel-packed column model KF-805 (Shodex). Number-average molecular weight (*M_n_*) of polymers was determined by the proton ratio of CH_2_ of PEO to CH_2_ of C18. Weight-average molecular weights (*M*_w_) of polymers were determined by using *M_n_* from ^1^H-NMR and polydispersity from GPC measurements. Critical micelle concentration (CMC) was determined by fluorescence probe method using sodium 8-anilino-1-naphthalene sulfonate (ANS-Na). Aqueous solutions of amphiphilic polymers were in the range of concentration from 1.0 × 10^−4^ to 1.0 mg/mL, in which ANS-Na was contained at the concentration of 2.0 × 10^−5^ M. The fluorescence spectra of ANS-Na from 400 to 600 nm were recorded on a Hitachi F-4500 spectrometer (Tokyo, Japan) with 390 nm of excitation wavelength. Fluorescence intensity at 490 nm was monitored to determine CMC. Concentration dependence of fluorescence spectra and intensity at 490 nm of aqueous (PEG2k)_2_-C18 solution containing ANS-Na were shown in Figure 1 as a typical example. The concentration, at which the slope of concentration dependence of the intensity at 490 nm was drastically changed, was defined as CMC. Aggregation numbers (*N_agg_*) of micelles were determined by field flow fractionation coupled with multiangle light scattering (FFF-MALS) using an Eclipse 3+ separation system with a Dawn Heleos II MALS detector (Wyatt Technology Europe GmbH, Dernbach, Germany). A Wyatt channel (Eclipse 3 channel LC) was used, which had a tip-to-tip length of 17.4 cm and a nominal thickness of 250 μm and a membrane (Nadir cellulose membrane 10 kDa LC) was attached to the bottom of the channel. The *N_agg_* of polymer micelles, which is the number of polymers in one micelle, was determined by dividing the weight average molar masses of the micelles obtained from Zimm plots of FFF-MALS by *M_w_* of polymers.

### 2.9. Small-Angle X-ray Scattering (SAXS)

For structural analyses of polymer micelles, SAXS measurements were performed at BL-40B2 station of SPring-8, Hyogo, Japan. Aqueous micelle solutions were injected into 2 mm *ϕ* quartz tubes and placed in the SAXS apparatus. A 30 x 30 cm^2^ imaging plate (Rigaku R-AXIS VII, Tokyo, Japan) was placed 1 m from the sample. The wavelength of the incident X-ray (*λ*) was adjusted to 0.10 nm. This setup provided a *q* range of 0.1–4 nm^−1^, where *q* is the magnitude of the scattering vector defined as *q* = (4π/*λ*)sin*θ* with a scattering angle of 2*θ*. The X-ray transmittance of the samples was determined by using ion chambers placed in front of and behind the sample. The 2-dimensional SAXS images were converted to one dimensional SAXS profiles of SAXS intensity *I*(*q*) vs. *q*.

## 3. Results and Discussion

**Figure 2:**
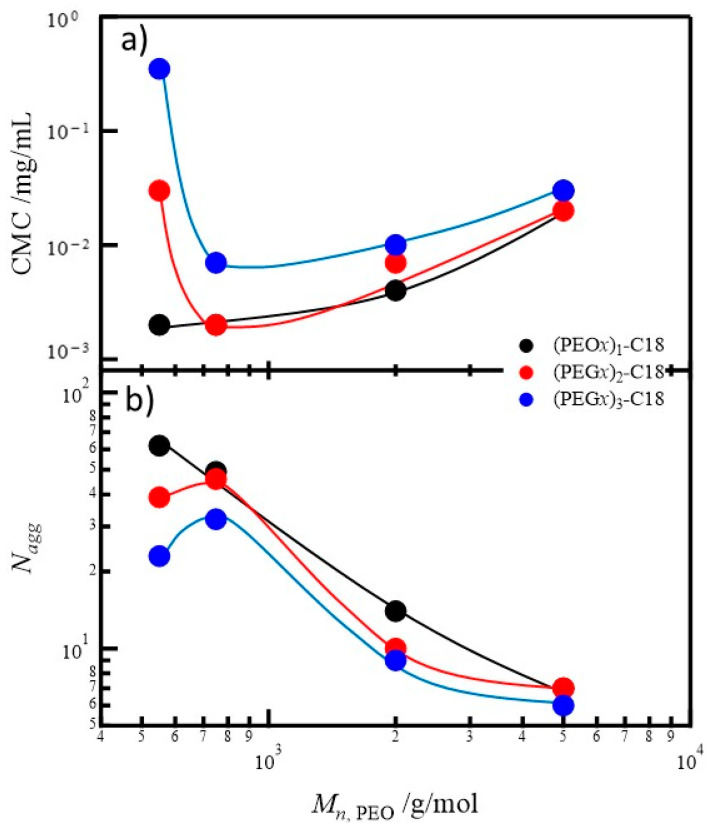
*M_n_*_,PEO_ dependence of CMC (a) and *N_agg_* (b) of aqueous (PEO*x*)*_y_*-C18 solutions.

The *M_n_*, *M_w_*, CMC and *N_agg_* of synthesized amphiphilic polymers were summarized in Table 1, and CMCs and *N_agg_*s were plotted against *M_n_* of PEO (*M_n,_*_PEO_) in Figure 2. As described above, the components of all polymers are the same and the only differences between polymers are the molecular architectures. Therefore, the differences in self-assemblies of these polymers should be derived from the differences of molecular architectures. The CMC of PEO-C18 is monotonically increased with increasing *M_n,_*_PEO_. In the case that *M_n,_*_PEO_ ≥ 750, (PEO*x*)_2_-C18 and (PEO*x*)_3_-C18 show similar manner of *M_n,_*_PEO_ dependence of CMC. These results can be explained due to increment of hydrophilicity with increasing *M_n,_*_PEO_ and number of PEO chain. Contrary to this tendency, CMCs of (PEO550)_2_-C18 and (PEO550)_3_-C18 are much higher than those of (PEO750)_2_-C18 and (PEO750)_3_-C18, respectively, although CMC of (PEO550)_1_-C18 follows the general trend of *M_n,_*_PEO_ dependence of CMC. In *M_n,_*_PEO_ dependences of *N_agg_*, (PEO550)_2_-C18 and (PEO550)_3_-C18 also show peculiarity. As shown in Figure 2, regardless of the number of PEO chain, *N_agg_*s of micelles are simply decreased with increasing *M_n,_*_PEO_, except for (PEO550)_2_-C18 and (PEO550)_3_-C18. Therefore, it is considered that the unique associating behavior in the star-like amphiphilic polymers having multiple hydrophilic PEO chains should remarkably appear in the region where the chain length of PEO is relatively short. This suggests that structures of micelles of (PEO*x*)_2_-C18 and (PEO*x*)_3_-C18 should drastically change between 550 and 750 g/mol of *M_n_*_,PEG_. Thus, SAXS measurements were carried out for (PEO*x*)*_y_*-C18 micelles.

**Figure 3:**
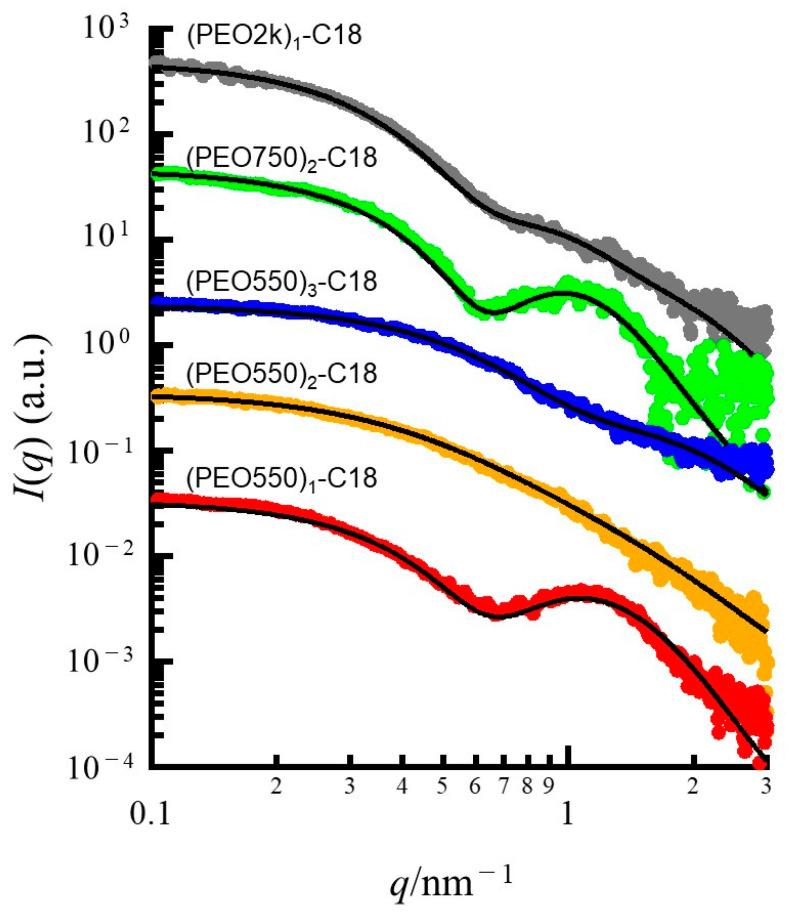
Small-angle X-ray scattering (SAXS) profiles of polymer micelles of (PEO2k)_1_-C18, (PEO550)_1_-C18, (PEO750)_2_-C18, (PEO550)_2_-C18, and (PEO550)_3_-C18. (PEO2k)_1_-C18, (PEO750)_2_-C18 and (PEO550)_3_-C18 are the almost same volume fraction of PEG in one (PEO*x*)*_y_*-C18.

Figure 3 compares SAXS profiles of (PEO*x*)*_y_*-C18 micelles. (PEO2k)_1_-C18, (PEO750)_2_-C18 and (PEO550)_3_-C18 are approximately equal mole fractions of PEO in (PEO*x*)*_y_*-C18, while (PEG550)_1_-C18, (PEG550)_2_-C18 and (PEG550)_3_-C18 are equal chain length of PEO. When *q* < 0.2 nm^−1^, *I*(*q*)s of all samples are almost independent of *q*. This means polymer micelles in this study form spherically symmetric shapes. In addition, this means there are no secondary aggregations of polymer micelles. On the other hand, SAXS profiles in *q* > 0.5 nm^−1^ show significant differences depending on the molecular structures. The SAXS profiles in this region reflect the internal composition distribution in the polymer micelles. To elucidate the origin of such differences in the SAXS profiles, numerical analyses using theoretical scattering function must be required. The solid lines in Figure 3 are theoretical SAXS curves calculated by the following core–corona model [16,17,18,19,20].

(1) $$\begin{array}{ll} P\left(q\right)= & N_{agg}{}^{2}\beta_{core}{}^{2}F_{core}{}^{2}\left(q\right)+N_{agg}\beta_{core}{}^{2}F_{chain}\left(q\right)\\ & \;\;\;+2N_{agg}{}^{2}\beta_{core}\beta_{chain}S_{core-chain}\left(q\right)+N_{agg}(N_{agg}\\ & \;\;\;-1)\beta_{chain}{}^{2}S_{chain-chain}\left(q\right) \end{array}$$

where *β_core_* and *β_corona_* are excess scattering lengths of hydrophobic core and PEO chain, respectively. *β_core_* = *V_core_*(*ρ_core_*−*ρ*_0_) and *β_coroae_* = *V_corona_*(*ρ_corona_*−*ρ*_0_), where *ρ_core_*, *ρ_corona_* and *ρ*_0_ are electron densities of core, PEO and solvent, respectively, and *V_core_* and *V_corona_* are volumes of core and corona, respectively. Here, we used 357 e^−^/nm^3^ for corona and 333 e^−^/nm^3^ for solvent. Thus, *V_core_*, *V_corona_*, and *ρ_coron_* are the unknown parameters. *F_core_*(*q*) is scattering amplitude of hydrophobic core regarded as sphere given by the following equation.

(2) $$F_{core}\left(q\right)=\frac{3\left[\sin\left(qR_{c}\right)-qR_{c}\cos\left(qR_{c}\right)\right]}{{\left(qR_{c}\right)}^{3}}\exp\left(-\frac{q^{2}\sigma^{2}}{2}\right)$$

where *R_c_* is radius of core and *σ* is thickness of interface between core and corona. The *R_c_* gives *V_core_*. We use the Debye function for *F_chain_*(*q*) given by the following equation [20].

(3) $$F_{chain}\left(q\right)=\frac{2\left[\exp\left(-q^{2}R_{g}{}^{2}\right)-1+q^{2}R_{g}{}^{2}\right]}{{\left(q^{2}R_{g}{}^{2}\right)}^{2}}$$

where *R_g_* is the radius of gyration of PEO. The *R_g_* gives the *V_corona_*. The third term in Equation (1) is the cross term between hydrophobic core and corona given by the following equation.

(4) $$S_{core-chain}\left(q\right)=F_{core}\left(q\right)\times A_{chain}\left(q\right)$$

where *A_chain_(q)* is the scattering amplitude of PEO chain given by the following equation.

(5) $$A_{chain}\left(q\right)=\frac{4\pi \int \rho_{chain}\left(r\right)\frac{\sin\left(qr\right)}{qr}r^{2}dr}{4\pi \int \rho_{chain}\left(r\right)r^{2}dr}\exp\left(-\frac{q^{2}\sigma^{2}}{2}\right)$$

The S_chain-chain_ is the cross term between different chains within the corona layer given by the following equation.

(6) $$S_{chain-chain}\left(q\right)=A_{chain}{\left(q\right)}^{2}$$

The P’(0) is the scattering of PEO chain at q = 0. Thus, by using N_agg_, R_c_, R_g_, and ρ_corona_ as adjustable parameters, we performed the fitting analyses for the experimental SAXS data to yield the smallest residues. Here, the N_agg_s from FFF-MALS were used as the initial values on N_agg_s in the fitting procedures. The solid lines are the best-fit results of numerical calculations. Table 2 summarizes the characteristics of (PEOx)_y_-C18 micelles obtained from the numerical calculations. Since the N_agg_s obtained from SAXS analyses well agree with those from FFF-MALS, the numerical analyses for SAXS by the core–corona model are reliable enough. From the results of numerical analyses, R_core_ and thickness of corona layer of (PEOx)_y_-C18 micelles are obtained.

**Figure 4:**
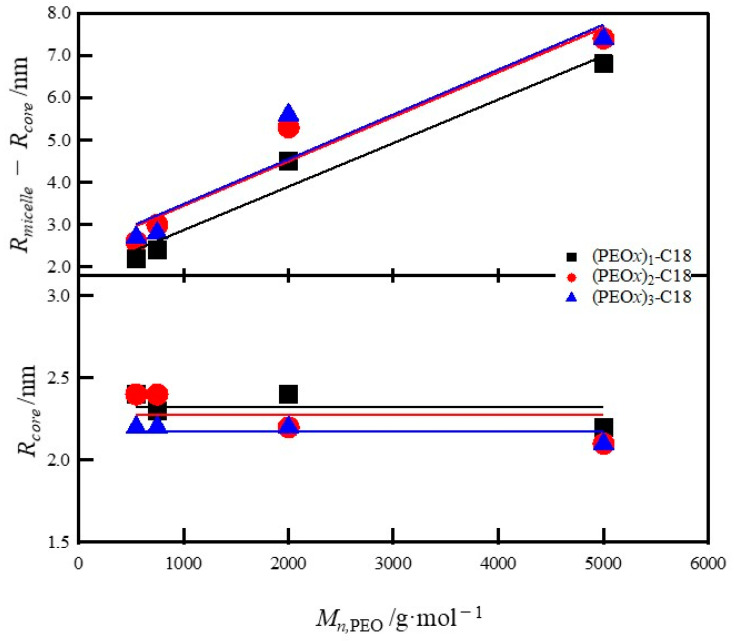
*M_n_*_,PEO_ dependence of *R_core_*s and thickness of corona of (PEO*x*)_y_-C18 micelles.

Figure 4 shows plots of *R_core_* and thickness of corona layer against the *M_n_*_,PEG_. *R_core_*s are almost constant regardless of *M_n_*_,PEO_ and molecular architecture because stearyl group is employed as hydrophobic chains for all (PEO*x*)*_y_*-C18. In addition, the thicknesses of the corona layers become predictably thick as the *M_n_*_,PEO_ increases. Moreover, the slopes of *M_n_*_,PEO_ dependence of the corona thickness are almost the same even if the molecular architecture is different. Therefore, significant specificity owing to differences of molecular architecture does not appear in the *M_n_*_,PEO_ dependences of *R_core_* and corona thickness. However, the corona thicknesses of (PEO*x*)_2_-C18 and (PEO*x*)_3_-C18 micelles always thicker than that of (PEO*x*)_1_-C18 micelles. This means that the characteristic feature of the (PEO*x*)_2_-C18 and (PEO*x*)_3_-C18 micelles derived from the differences in molecular architecture only appears in the difference in the thicknesses of corona layers in (PEO550)_2_-C18 and (PEO550)_3_-C18 micelles. This result is consistent with the results obtained from the *M_n_*_,PEO_ dependences of CMC and *N_agg_* as shown in Figure 2. The difference in the number of PEO chains should affect the number density of PEO chains or the occupied area of one PEO chain on the core–corona interface of (PEO*x*)_y_-C18 micelles. Since these parameters indicate the degree of crowding of hydrophilic chains on the interface [21,22,23,24], they are significantly important to characterize the polymer micelles.

**Figure 5:**
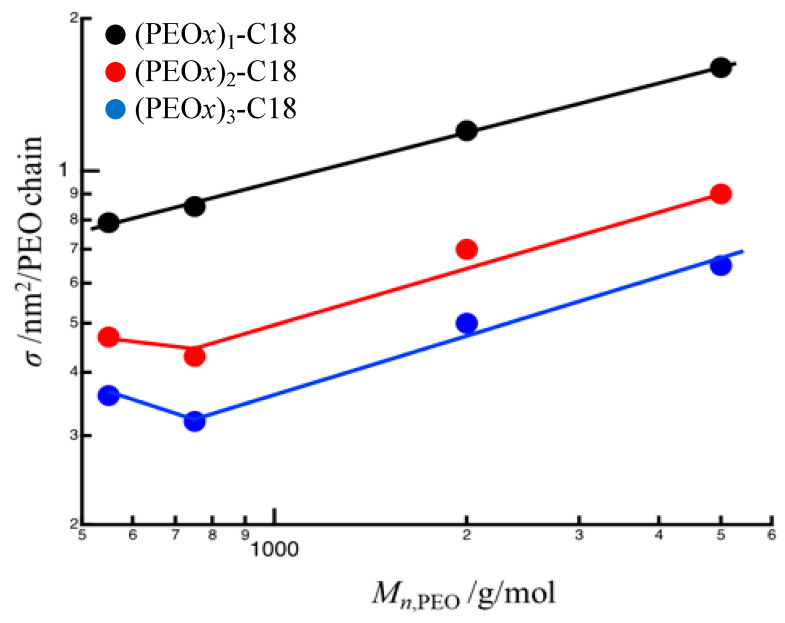
Occupied area of one PEO chain of (PEO*x*)*_y_*-C18 on the interface of hydrophobic core and corona layer in (PEO*x*)*_y_*-C18 micelles.

**Figure 6:**
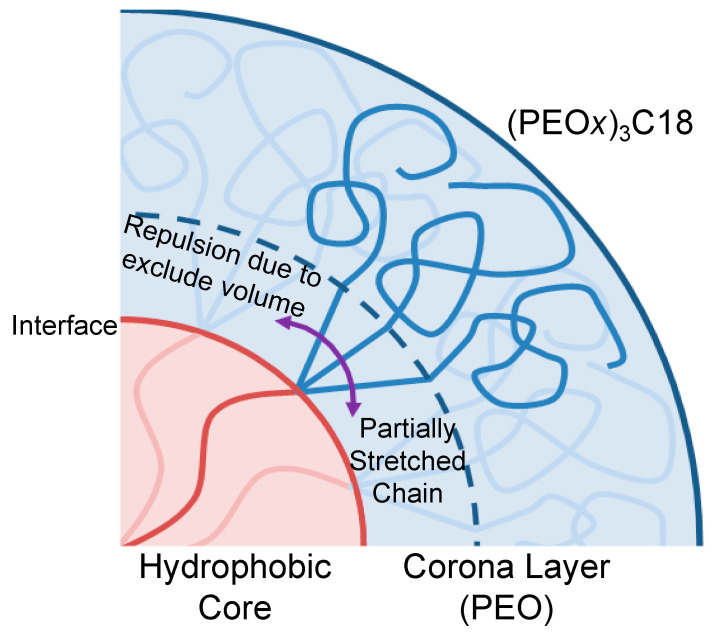
Schematic representation of conformation of PEO chains in (PEO*x*)_3_-C18 micelles.

Figure 5 shows the occupied area of one PEO chain (*σ*) defined as 4*πR_core_*/*N_agg_* against *M_n_*_,PEO_. The *σ* decreases as the number of PEO chains in one (PEO*x*)*_y_*-C18 molecule increases. That is, the larger the number of PEO chains in one (PEO*x*)*_y_*-C18 molecule, the more crowded the PEO chains at the core–corona interface in (PEO*x*)*_y_*-C18 micelles. Therefore, it is considered that the partial PEO chains of (PEO*x*)_2_-C18 and (PEO*x*)_3_-C18 near the core–corona interface locally take an unfavorable conformation due to the excluded volumes of PEO as schematically shown in Figure 6. When the PEO chains are long enough, most parts of the PEO chains can take stable conformation [18,20,21]. Since the effect of the PEO crowding near the interface in (PEO*x*)_2_-C18 and (PEO*x*)_3_-C18 micelles is negligible, these polymers should show similar associating behavior to (PEO*x*)_1_-C18. However, as the chain length of PEO becomes shorter, the PEO crowding near the interface have significant effect on the micelle formation of (PEO*x*)_2_-C18 and (PEO*x*)_3_-C18. Since the boundary *M_n_*_,PEO_ is located between 550 and 750 g/mol, the curvatures of the interfaces become large and the *N_agg_*s become small in (PEO550)_2_-C18 and (PEO550)_3_-C18 micelles in order to eliminate the unfavorable conformation of PEO chains near the core–corona interface.

## 4. Conclusions

Here, we showed associating behavior of (PEO*x*)_y_-C18, which is a star-like amphiphilic polymer with plural PEO chains. We found characteristic *M_n_*_,PEO_ dependence of *N_agg_* and CMC of (PEO*x*)_2_-C18 and (PEO*x*)_3_-C18 micelles in the low *M_n_*_,PEO_ region. SAXS measurements revealed that the occupied area of one PEO chain on the interface between hydrophobic core and corona in (PEO*x*)_2_-C18 and (PEO*x*)_3_-C18 micelles were much narrower than that in (PEO*x*)_1_-C18 micelles. This result indicated the partial PEO chains in (PEO*x*)_2_-C18 and (PEO*x*)_3_-C18 micelles locally took an unfavorable conformation near the interface between hydrophobic core and corona layer because of crowding of PEO chains. The effect of such unfavorable conformation of partial PEO chains near the interface on the formation of polymer micelles became significant as *M_n_*_,PEO_ decreased. Therefore, the characteristic *M_n_*_,PEO_ dependence of *N_agg_* can be explained by the formation of unfavorable conformation of PEO chains near the core–corona interface in (PEO*x*)_2_-C18 and (PEO*x*)_3_-C18 micelles owing to the crowding of PEO chains near the interface.

## Figures and Tables

**Table 1:** Molecular weight, CMC and aggregation number (*N_agg_*) of micelles of (PEO*x*)_y_-C18s.

Polymers	*M_n_*/g/mol	*M_w_*/g/mol	*M_w_*/*M_n_*	CMC/mg/mL	*N_agg_*
PEO550-C18	8.2 × 10^2^	9.5 × 10^2^	1.2	0.001	54
PEO750-C18	1.0 × 10^3^	1.1 × 10^3^	1.1	0.002	44
PEO2k-C18	2.3 × 10^3^	2.4 × 10^3^	1.04	0.005	14
PEO5k-C18	5.3 × 10^3^	5.4 × 10^3^	1.02	0.03	7
(PEO550)_2_-C18	1.5 × 10^3^	1.7 × 10^3^	1.2	0.03	32
(PEO750)_2_-C18	1.9 × 10^3^	2.2 × 10^3^	1.2	0.002	41
(PEO2k)_2_-C18	4.4 × 10^3^	4.8 × 10^3^	1.1	0.007	9
(PEO5k)_2_-C18	1.0 × 10^4^	1.1 × 10^3^	1.1	0.02	8
(PEO550)_3_-C18	2.0 × 10^3^	2.4 × 10^3^	1.2	0.3	16
(PEO750)_3_-C18	2.6 × 10^3^	3.0 × 10^3^	1.2	0.005	29
(PEO2k)_3_-C18	6.4 × 10^3^	7.0 × 10^3^	1.1	0.01	9
(PEO5k)_3_-C18	1.5 × 10^4^	1.6 × 10^4^	1.1	0.03	6

**Table 2:** Characteristics of (PEO*x*)*_y_*-C18 micelles obtained from SAXS analyses.

Polymers	*R_core_/nm*	*R_micelle_* /nm	*R_g,_*_PEG_/nm	*ρ_core_*/e^−^/nm^3^	*N_agg_*
PEO550-C18	2.4	4.6	1.2	320	62
PEO750-C18	2.3	4.7	1.4	323	44
PEO2k-C18	2.4	6.9	2.6	331	14
PEO5k-C18	2.2	9.0	3.9	332	7
(PEO550)_2_-C18	2.4	5.0	1.1	329	39
(PEO750)_2_-C18	2.4	5.4	1.4	324	46
(PEO2k)_2_-C18	2.2	7.5	2.8	327	10
(PEO5k)_2_-C18	2.1	9.5	3.9	331	7
(PEO550)_3_-C18	2.2	4.9	0.5	326	23
(PEO750)_3_-C18	2.2	5.0	1.7	319	31
(PEO2k)_3_-C18	2.2	7.8	3.2	330	9
(PEO5k)_3_-C18	2.1	9.5	3.9	333	6

## Data Availability

The data presented in this study are available on request from the corresponding author.
